# Spatial Distribution Characteristics of the Rural Tourism Villages in the Qinghai-Tibetan Plateau and Its Influencing Factors

**DOI:** 10.3390/ijerph19159330

**Published:** 2022-07-30

**Authors:** Jianwei Qi, Yayan Lu, Fang Han, Xuankai Ma, Zhaoping Yang

**Affiliations:** 1 Xinjiang Institute of Ecology and Geography, Chinese Academy of Sciences, Urumqi 830011, China; qijianwei16@mails.ucas.ac.cn (J.Q.); luyayan16@mails.ucas.ac.cn (Y.L.); hanfang@ms.xjb.ac.cn (F.H.); maxuankai20@mails.ucas.ac.cn (X.M.); 2 University of Chinese Academy of Sciences, Beijing 100049, China

**Keywords:** rural tourism, spatial distribution, influencing factor, geographic detector, Qinghai Tibetan plateau

## Abstract

The development of rural tourism (RT) has great significance in reducing poverty and achieving rural vitalization. Qinghai-Tibetan Plateau (QTP) is a depressed area with rich RT resources due to its unspoiled nature and diverse culture. For future sustainable development of RT in QTP, this paper analyzes the spatial distribution characteristics and its influencing factors of RT villages using various spatial analysis methods, such as nearest neighbor index, kernel density estimation, vector buffer analysis, and geographic detectors. The results show the following. First, the RT villages present an agglomeration distribution tendency dense in the southeast and spare in the northwest. The inter-county imbalance distribution feature is obvious and four relatively high-density zones have been formed. Second, the RT villages have significant positive spatial autocorrelation, and the area of cold spots is larger and of hot spots is smaller. Third, the RT villages are mainly distributed with favorable topographic and climate conditions, near the road and water, around the city, and close to tourism resources. Fourth, the spatial distribution is the result of multifactor interactions. Socio-economic and tourism resource are the dominant factor in the mechanism network. Fifth, based on the above conclusions this study provides scientific suggestions for the sustainable development of the RT industry.

## 1. Introduction

Tourism is generally considered one of the world’s most important economic sectors, enabling socio-economic development, including job creation, poverty reduction, driving prosperity, and significant positive social impacts such as providing unique opportunities to women, minorities, and youth [1]. Moreover, tourism will play an important role in the recovery of national economies and global trade, especially for developing economies in the post-COVID-19 situation [2]. Rural tourism (RT) is a form of tourism based on a rural geographic location and traditional rural production methods and culture [3]. The location of RT should have a low population density and dominate by agriculture and forestry, and the tourism activity of RT include nature-based activities, agriculture, rural lifestyle or culture, angling, and sightseeing [4,5]. Due to the characteristics of RT activities, RT is believed to face more opportunities during the post-COVID-19 era as tourists seek to escape from densely populated urban centers to naturally remote areas [6].

RT is regarded as an essential engine for driving rural development, taking account of the economy, society, and environment [7]. For instance, RT positively and significantly affects villagers’ income and happiness [8,9], maintains livelihood sustainability, preserves the biodiversity of the rural inhabitants, and prevents the trend of depopulation in rural areas [10,11,12]. The positive change brought by RT has been proven in many developed and developing countries. Specifically, RT development not only achieves considerable economic benefits, which assist in development but also creates a good basis for further tourism activities with marketing and promotion for the community [13,14,15]. In China, RT is an emerging and effective catalyst that promotes industrial restructuring, agricultural development, and the upgrading of rural areas [16]. Especially after 2018, China announced the implementation of the Rural Revitalization Strategy (RRS) and proposed to build the countryside into a regional complex with natural, social, and economic characteristics [17,18]. In 2019, the sector of RT recorded 3.2 billion person-visits, generating over 850 billion yuan in revenue, a year-on-year increase of 6.3% [19].

The study of RT has been a key research area over the last few decades. The developed countries majorly contributed from 1980 to 1999, and the developing countries contributed to the last two decades. Moreover, the study area is also different due to their unlike development and growth contexts [20,21]. QTP is an area where villages occupy an important position in terms of history, population, and space in regional society [22]. According to the National Population Census of 2020, the rural population accounts for 52.63% of the total QTP population, which is 16.52% higher than the average at the national level. QTP, a remote and underdeveloped area, has limited development options because of various disadvantages such as lower level of economic and undeveloped infrastructure, so developing tourism becomes a high priority choice in QTP [23]. In this context, promoting RT should be strongly focused on QTP, as RT offers useful opportunities to solve the development problems of QTP. However, the only two studies related to RT in the QTP only explored the relationship between RT and agricultural production and the influence of social media on resident participation in RT [24,25]. Understanding the RT’s development patterns, mechanisms, and influencing factors are crucial for scientific regional tourism planning and management, which can solve problems such as improper site selection, layout dispersion, and market competition disorder [26,27]. Hence, studying RT spatial characteristics, influencing factors, and driving mechanisms are necessary for the sustainable development of RT from different perspectives.

With the development of Geographic Information System (GIS), and the wide application of spatial big data, research on the spatial patterns of RT villages in China has made significant progress. Wang [28], Zhan [29], and Lei [30] using GIS and spatial analysis with mathematical statistics including nearest neighbor index, kernel density estimation, and spatial autocorrelation, to analyze the RT villages on a national scale, find that RT villages in China show an unbalanced spatial distribution pattern, mainly distributed in the eastern region of China, southeast of Hu line. The inter-provincial spatial density stratification feature is obvious. Xu [31], Zhang [32], Shen [33], and Yu [34] analyze the RT villages’ spatial patterns in Shaanxi, Liaoning, Jiangsu, and Hubei, and give suggestions for layout optimization and sustainable development. Prior research has thoroughly investigated the spatial character of RT villages in China; however, there remain some shortcomings. First, these studies focus on the spatial scale of administrative boundaries, including the whole country or single province, lacking study from the natural zones scale. Second, most of the studies at the provincial scale were conducted in the relatively developed regions of China, neglecting the regions with large rural areas where development of RT is a development tool such as in western China. Third, existing research lacks the analysis using county as the basic unit. The county is China’s basic administration and economic unit, representing the unique regional resources and industrial characteristics. Thus, taking the county as the basic unit can better reveal the spatial character. QTP is one of the ethnic minorities-concentrated regions and the remotest areas in China’s regional society. The area retains the rural features with a high value of rurality and has unique natural geographical conditions [35]. Therefore, the research of RT villages in the QTP would be of great significance for the response to the Chinese government’s making QTP a world tourist destination and green development strategy.

In terms of the influencing factors of RT villages, some authors have recognized the influence of a single factor and discussed the influence of a certain factor on RT development, such as the influence of government [36], environment [37], and community participant [38]. Some authors have driven the further development of influence factors from single factor domine influence to multiple factor influence. For example, An W. believes the influence factor of RT is staff hospitality, outdoor activities, additional facilities, and location [39]. From the experience of America, environmental facilities, residents, and economic spillover are the main factors of RT [40]. In South Korea, the economy, social culture, management, and the environment play important roles in the development of RT [41]. Streimikiene thinks RT is integrated with the economic, social, cultural, human resources, and local structure. There is a strong correlation between these factors, but the individual factors and their influence on the development of RT have been explored just fragmentally [42]. In short, previous studies have emphasized the importance of a comprehensive analysis of the of RT villages’ influencing factors both social and natural. We can supplement existing research in the following ways: First, Geodetector is a useful statistical method to detect spatial differentiation and reveal the driving factors, it has been applied to analyze the influencing factors of tourism demand, tourism ecological security, and other tourism-related research [43,44]. Using Geodetector is a meaningful attempt at the existing method of measuring influencing factors. Second, QTP has unique natural environment characteristics distinctive from other regions in the world, caused by high altitude, thin air, complex landforms, and fragile ecological environment [45]. To our knowledge, no prior studies have examined the influence factor in a similar area. We analyze the influence of physical and socio-economic factors to understand the underlying mechanism of RT villages in QTP, and can make optimization suggestions for the sustainable development of RT.

Therefore, this study has three main research objectives: (1) Using series GIS spatial tools to analyze the spatial character of RT villages at the integrated geographical scale; (2) using Geodetector and mathematical statistics to identify the influencing factors of RT villages in QTP; (3) proposing sustainable development measures and suggestions for the government to achieve the goal of World Tourist Destination and Rural Revitalization for QTP.

## 2. Materials and Methods

### 2.1. Study Area

**Figure 1:**
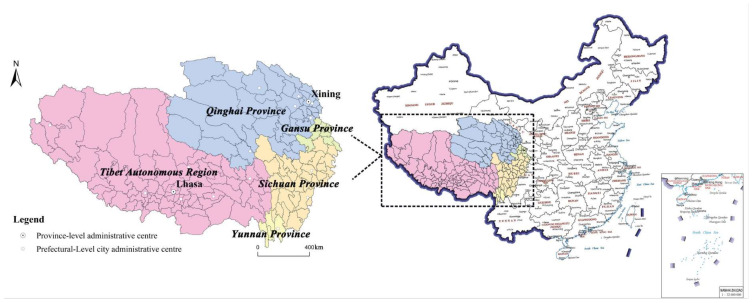
The location of study area (Map Approval Number: GS (2019)1686).

The QTP with an average elevation exceeding 4500 m and an area of 2.5 × 10^6^ km^2^, is the world’s highest and largest plateau, known as the “Earth’s Third Pole” and “Roof of the World”. The part of QTP in China located in the southwest of China covers most of the Tibet Autonomous Region (TAR), most of Qinghai province, northwestern Yunnan province, western Sichuan province, and southern Gansu province. As shown in Figure 1, this study includes 161 counties (74 in TAR, 44 in Qinghai, 5 in Yunnan, 31 in Sichuan, and 7 in Gansu). QTP is the less developed and low population density area, which occupies 23% of the country’s land area but only has 0.87% of the population and 0.64% of the GDP. QTP is rich in tourism resources, including unspoiled natural environments, historical and cultural monuments, and sustained folk customs and festivals. Such as the world’s highest mountain (Chomolungma and its surrounding mountains); the deepest canyon (Yarlung Zangbo Daxiagu); and the World Cultural Heritage Potala Palace, Jokhang Temple, and Norbulingka. The tourism industry in QTP has been developing rapidly in recent years due to the strategy of making QTP a world tourist destination proposed by the Tibet Work Forum of The CPC Central Committee. The tourist number was 175.7 million in 2019 (4.5 times the number in 2010), and the tourism income amounted to RMB 190.5 billion in 2019 (4.2 times the number in 2010).

### 2.2. Data Source and Processing

We use various RT villages lists proposed by different departments of the Chinese government, including National Tourism Key Village, Distinctive Tourist Village, Beautiful Leisure Village, Traditional Village, Minority Ethnic Village and Forest Village. Lists are taken from each department’s website. National Tourism Key Village and Distinctive Tourist Village were from the Ministry of Culture and Tourism of China (https://www.mct.gov.cn/, accessed on 15 June 2022); Beautiful Leisure Village was from the Ministry of Agriculture of Rural Affairs of China (http://www.moa.gov.cn/, accessed on 15 June 2022); Traditional Village was from the Ministry of Housing and Urban-Rural Development of China (https://www.mohurd.gov.cn/, accessed on 15 June 2022); Minority Ethnic Village was from the National Ethnic Affairs Commission of China (https://www.neac.gov.cn/, accessed on 15 June 2022); Forest Village was from the National Forestry and Grassland Administration (http://www.forestry.gov.cn/, accessed on 15 June 2022). After sorting and screening, excluding duplicate villages, a total of 640 village lists were obtained. Based on the list, we utilize Google Earth to obtain the geographical coordinates of each village and to build a point vector database.

The digital elevation model (DEM) with a resolution of 90 m × 90 m data was derived from the Google Earth Engine online dataset [46]. Meteorological data were from the National Tibetan Plateau Data Center (https://data.tpdc.ac.cn/, accessed on 15 June 2022). Basic geographic data (e.g., administrative divisions, transportation, government locations, river) were from the China National Basic Geographic Information Center (www.ngcc.cn/ngcc/, accessed on 15 June 2022). GDP and population data were from the statistical yearbooks of the relevant Counties.

### 2.3. Research Method

To analyze the spatial distribution character of RT villages, first, we use nearest neighbor index to determine the spatial type; second, we use geographic concentration index and Lorenz curve to evaluate the equilibrium degree and agglomeration degree; third we use kernel density estimation to reveal the distribution density; last, we use Moran’s I and Getis-Ord $G_{i}^{*}$ to measure spatial autocorrelation of the area in global and regional. These methods are widely used in the study of spatial statistics to analyze spatial distribution. In terms of the influencing factors, as summarized in the introduction, the spatial distribution of RT villages is influenced by multiple factors and should be analysis comprehensive from the perspectives of both natural conditions and human factors. Concerning relevant studies, the analysis of natural factors mainly includes topography, climate, and hydrology. Moreover, we analyzed the effect of oxygen content as a climate factor specific to the QTP. The analysis of human factors includes traffic condition, socio-economic and tourism resource. We analyzed the effect of culture heritage as QTP has rich culture resource. Finally, we select 13 evaluation indexes and the geographical proxy variables of influencing factors, as shown in Table 1. Then we mainly use Geodetector, which has an advantage in measuring multi-level spatial differentiation and is supplemented by overlay analysis, buffer analysis, and correlation analysis, to interpret and analyze the influence factors in depth. These methods are implemented through ArcGIS 10.5 and R to analysis and achieve a visual display of the data.

#### 2.3.1. Nearest Neighbor Index

Nearest neighbor index is a method to measure the spatial distribution type of point elements, through analysis of the proximity in geographical space by the nearest neighbor distance [47]. The calculation formulas are as follows:

(1) $$R=R_{i}/R_{e}=\frac{1}{n}\overset{n}{\underset{i=1}{\sum }}d_{i}\left(s_{i}\right)\times \frac{1}{2\sqrt{n/A}}$$

where *R* is the nearest neighbor index, $R_{i}$ represents the theoretical nearest distance, and $R_{e}$ represents the actual nearest distance, $d_{i}\left(s_{i}\right)$ represents the distance of the RT villages to the nearest RT villages, *n* represents the number of RT villages in the QTP, *A* is the area of the QTP. If the index is less than 1, the distribution pattern exhibits agglomerative; if the index is greater than 1, the trend is toward uniform.

#### 2.3.2. Geographic Concentration Index

Geographic concentration index is a method to measure the degree of spatial distribution and agglomeration of objects. The calculation formulas are as follows:

(2) $$G=\sqrt{\overset{n}{\underset{i=1}{\sum }}{\left(X_{i}/T\right)}^{2}}\times 100\%$$

where *G* is the index of geographic concentration, $X_{i}$ is the number of RT villages in the No.*i* county, *T* is the total number of RT villages in the QTP, *n* is the number of counties in QTP. *G* is between 0 to 100, the larger the *G*, the stronger the degree of spatial agglomeration.

#### 2.3.3. Global Moran’s I

Moran’s I is a measure of global spatial autocorrelation defined by Moran, based on the first law of geography [48]. Spatial autocorrelation means how one object is similar to another’s surrounding. The calculation formulas are as follows:

(3) $$\;\;Moran^{’}s\;I=n\frac{\sum_{i=1}^{n}\sum_{j=1}^{n}w_{ij}\left(x_{i}-\bar{x}\right)\left(x_{j}-\bar{x}\right)}{\sum_{i=1}^{n}\sum_{j=1}^{n}w_{ij}\sum_{i=1}^{n}{\left(x_{j}-\bar{x}\right)}^{2}}\;$$

where $x_{i}$ and $x_{j}$ represent the number of RT villages in No.*i* county and No.*j* county, $\bar{x}$ represents the average number of RT villages, $w_{ij}$ represent the spatial adjacent weight matrix of the counties, *n* represents the total number of counties. The values of Moran’s I range from −1 to +1, the value of +1 meaning strong positive spatial autocorrelation, to 0 meaning a random pattern, and to −1 indicating strong negative spatial autocorrelation.

#### 2.3.4. Geits-Ord $G_{i}^{*}$

Geits-Ord $G_{i}^{*}$ is a local spatial autocorrelation defined by Getis and Ord that describes how large the neighborhood of a given site is relative to the average neighborhood, focused on identifying the so-called “hotspots” and “coldspots” [49]. the calculation formula is as follows:

(4) $$G_{i}^{*}=\overset{n}{\underset{j=1}{\sum }}w_{ij}x_{j}/\overset{n}{\underset{j=1}{\sum }}x_{j}$$

where $w_{ij}$ represents the spatial adjacent weight matrix of the counties, *n* represents the total number of counties. $x_{j}$ represents the number of RT villages in No.*i* county and No.*j* county. If the result of $G_{i}^{*}$ is positive and significant, the area belongs to the hot spot area, and reflects high-value spatial agglomeration; otherwise, it belongs to the cold spot area and reflects low-value spatial agglomeration.

#### 2.3.5. Kernel Density Estimation

Kernel density estimation is a non-parametric technique for density estimation in which a known density function (the kernel) is averaged across the observed data points to create a smooth approximation. Kernel density estimation can reflect the distribution characteristics of samples by examining the regional spatial changes of sample point density. The results can identify the concentration and dispersion of the regional samples. The calculation formula is as follows:

(5) $$f\left(x\right)=\frac{1}{nh}\overset{n}{\underset{i=1}{\sum }}k\left(\frac{x-x_{i}}{h}\right)$$

where $f\left(x\right)$ is the kernel function, $x_{i}$ represents the coordinate position of the *i*-th RT villages, *k* is the threshold of the kernel function.

#### 2.3.6. Geodetector

Geodetector is a spatial statistics method to detect spatial heterogeneity and quantify the influence of driving factors by comparing the spatial consistency of independent variable distribution versus the geographical strata in which potential factors exist [50]. The influence power is measure by *q*, calculation formula is as follows:

(6) $$q=1-\frac{\sum_{h=1}^{L}N_{h}\sigma_{h}{}^{2}}{N\sigma^{2}}$$

where *L* is the number of classifications of factor, *N* is the number of units in whole region, $\sigma_{h}{}^{2}$ and $\sigma^{2}$ are the variance of the class *h* and the whole region. The *q* values range from 0 to 1, and the larger the q value is, the strongest the explanatory power of factor.

## 3. Results Analysis

### 3.1. Spatial Distribution Characteristics

**Figure 2:**
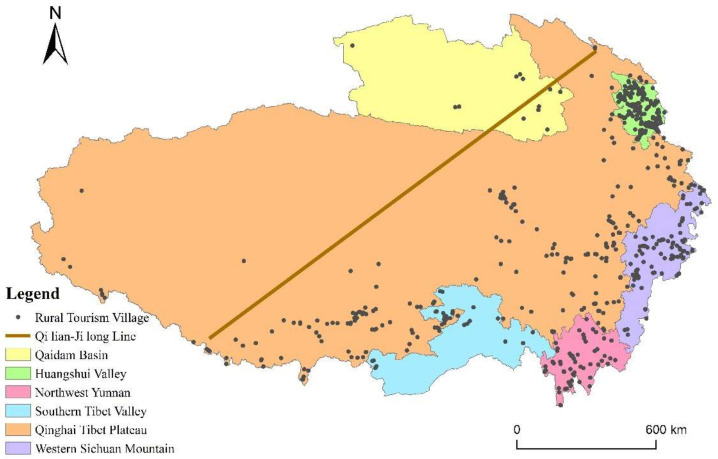
Spatial distribution of RT villages in QTP.

The RT villages in QTP showed a trend of aggregated distribution in space (Figure 2). Using nearest neighbor index to measure the spatial distribution type, the result shows that the actual closet distance is 15.62 km, the theoretical closet distance is 40.46 km, and the nearest neighbor index is 0.39. This indicates that RT villages in QTP are distributed in a state of concentration. From a macro perspective, the number and density of RT villages on the southeast side are significantly higher than those on the northwest side. This type of distribution is consistent with the “Qilian-Jilong Line”, an imaginary line that divides the QTP area into southeast and northwest parts with contrasting population densities. The area of the two parts is roughly the same, but the population ratio of the southeast and the northwest parts is 93:7 [51]. The population distribution type directly impacts the distribution type of RT villages, which is “Dense in southeast and sparse in northwest”. From the perspective of the human geographic sub-unit in QTP proposed by Fang et al. [52], the most densely distributed sub-unit is Huangshui Valley, with an average of 47 RT villages per 10,000 km^2^, and the most sparely distributed sub-unit is Qaidam Basin, with only 0.3 RT villages per 10,000 km^2^. 

**Figure 3:**
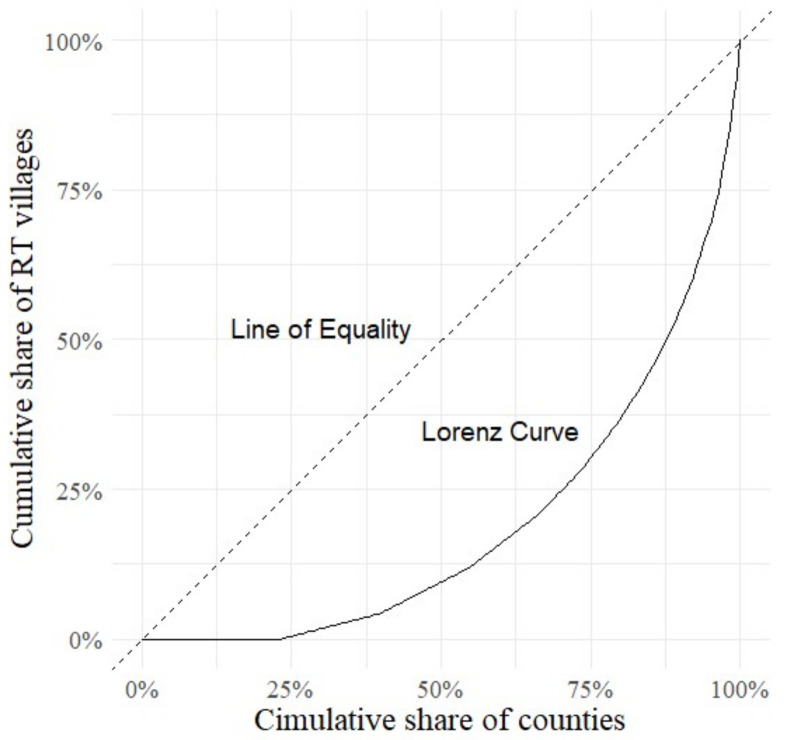
Lorenz curve of RT villages in QTP.

The RT villages in QTP present uneven spatial distribution features between counties. Using geographic concentration index to measure the degree of equilibrium of RT villages, the result shows that the geographic concentration index is 13.3%. If RT villages were evenly distributed in each county, the geographic concentration index would be 6.1%. Therefore, the distribution of RT villages shows a weak degree of concentration at the county scale. The Lorenz curve was plotted using the cumulative percentage of RT villages to further reveal the unevenness of the distribution. As shown in Figure 3, the Lorenz curves presented a typical concave form, indicating that RT villages have an unbalanced distribution between counties. The largest number of RT villages are in Xunhua County, with 39 RT villages, and there are 38 counties with no RT villages.

**Figure 4:**
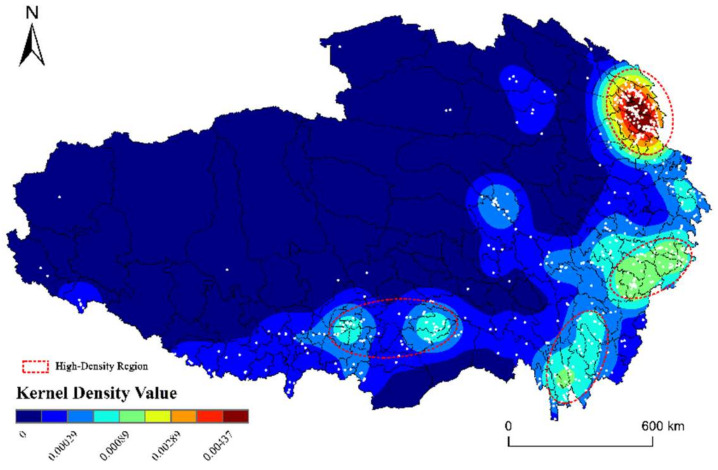
Kernel density map of RT villages in QTP.

The “Density Analysis” tool in ArcGIS is used to calculate the kernel density value and make the kernel density distribution map of RT villages in QTP (Figure 4). Based on the map, we can find four high-density regions in QTP. The first high-density region is the Huangshui Valley, covering the area of Xining city and Haidong city in Qinghai province. The highest kernel density value in this region reached 0.0043, which means there are 43 villages per 10,000 km^2^ in Huangshui valley. Huangshui valley locates at the northeast edge of QTP, is the transition area from the QTP to the Loess Plateau, with a relatively low-altitude, denser population distribution, and is an important area for the integration, exchange, and development of various ethnic groups [53]. Therefore, Huangshui Valley forms the highest kernel density value of QTP. The second high-density region is the west Sichuan Mountain, covering the area of Aba prefecture and Ganzi prefecture. The core density value of this region ranges from 0.0008–0.0021. This region has profound historical and cultural resources, such as cultural relics and intangible cultural heritage and has outstanding characteristics [54]. Historical and cultural resources are the main attractions of the RT villages in this region. The third high-density region is the Northwest Yunnan, also known as Shangri-La. Covering the area of Diqing prefecture. This region is famous for the novel “Lost Horizon”, a remote idyllic place with magnificent landscapes shrouded in mysticism [55]. The fourth high-density region is YNL River region, with Lhasa city and Linzhi city in TAR as the core. YNL River region is also named as the Yarlung Zangbo River, Nyangqu River, and Lhasa River region. This region accounts for about one-third of the total population of TAR, and has flatter land, superior natural conditions, and a better economic level than other regions in the TAR [56].

**Figure 5:**
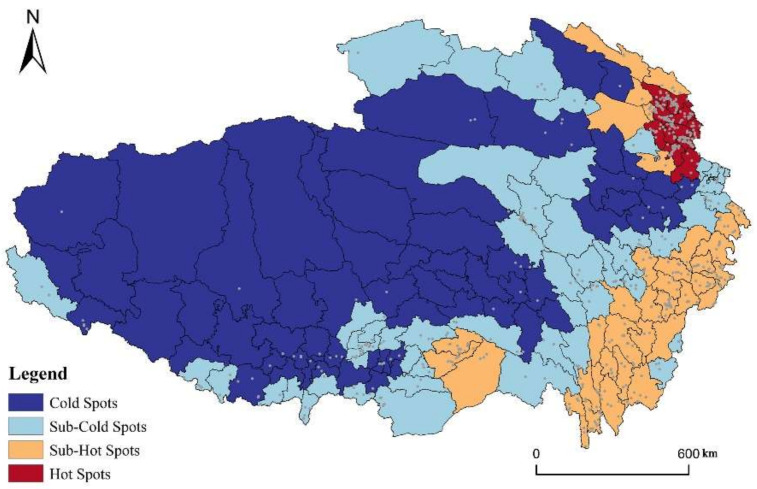
Spatial differentiation of cold and hot spots of RT villages in QTP.

Using “Global Moran’s I” tool in ArcGIS to detect the spatial autocorrelation of RT villages in QTP based on county locations and counts of RT villages per county. The result shows that the Moran’s index value is 0.268, indicating a positive spatial autocorrelation. The *z*-score is 9.36, higher than the 2.58 standard deviations, and the *p*-value is less than 0.01. Based on the combination of *z*-score and *p*-value, we can reject the null hypothesis: RT villages in QTP are Complete Spatial Randomness (CSR) at 99 percent confident level. We can draw a conclusion that the RT villages in QTP have a significant positive spatial autocorrelation. Further, “Geits-Ord $G_{i}^{*}$” tool in ArcGIS is used to explore the correlation index of counties, and the result was divided into hot spots, sub-hot spots, sub-cold spots, and cold spots through Jenks natural breakpoint method (Figure 5). The hot spots are the smallest, located in the Huangshui Valley. Chengdong, Datong, Hualong, and other 11 counties belong to Xining and Haidong city in Qinghai province, accounting for only 2.13% of the QTP’s total area. The cold spot has the largest area and includes 58 counties, mainly distributed in Rikaze city, Naqu city, Ali prefecture, Changdu city, and Shannan city in TAR, and Guoluo prefecture, and Haixi prefecture in Qinghai province, covering 59.1% of the QTP’s entire area. Sub-hot spots are mainly distributed around hotspots and the east edge of QTP. The area surrounding the hot spots is mainly 7 counties in Haibei prefecture and Hainan prefecture in Qinghai province. The counties on the east edge of QTP are mainly in Ganzi, Aba, and Diqing prefecture. The remaining counties belong to sub-cold spots, mainly arranged in the junction areas of the cold spots and sub-hot spots, and some isolated county south of TAR. Overall, the cold and sub-cold spots are significant areas for local spatial differentiation of RT villages in QTP, accounting for 86.1% of the total. The distribution areas of hot and sub-hot spots are relatively small, accounting for 13.9% of the total.

### 3.2. Influencing Factors 

#### 3.2.1. Topographic

QTP starts from the Himalayas in the south, the Kunlun Mountains and Qilian Mountains in the north, the Pamir Plateau and the Karakoram Mountains in the west, and connects with the western section of the Qinling Mountains and the Loess Plateau in the east and northeast. It is the first-tiered terraces of China’s topography, with a very complex topography and great variations. The terrain of the QTP is high in the west and low in the east, with rough edges and large internal fluctuations. The diverse and complex topography is the crucial physical characteristic that makes the QTP distinctive and is the fundamental factor in forming the pattern of social and economic development. 

**Figure 6:**
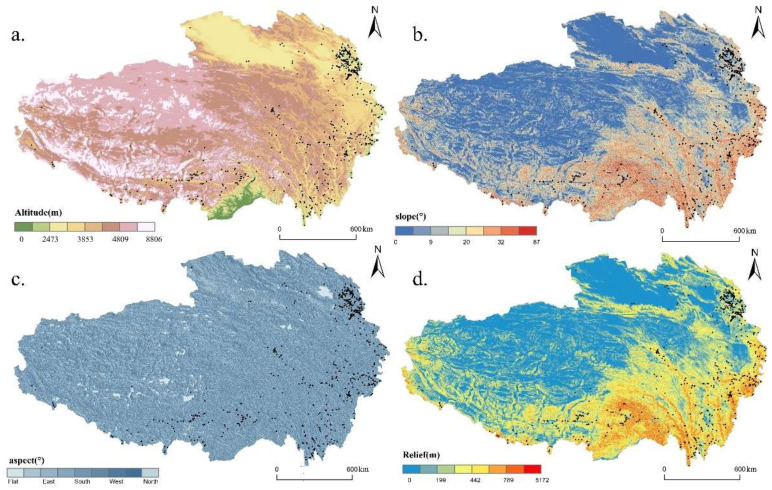
Spatial distribution of RT villages coupled with topographic: (**a**) altitude; (**b**) slope; (**c**) aspect; (**d**) relief.

**Figure 7:**
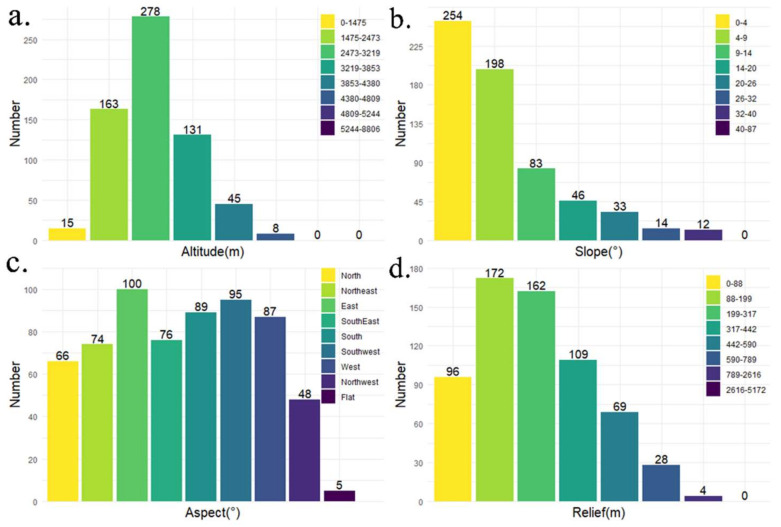
Statistics of RT villages in different topographic conditions: (**a**) altitude; (**b**) slope; (**c**) aspect; (**d**) relief.

Altitude directly impacts the site selection, size, and structure of village by influencing natural environmental factors such as climate, vegetation, and soil. As presented in Figure 6a, we reclassified QTP’s altitude through natural breaks methods and performed an overlay analysis with the distribution map of RT villages. As shown in Figure 7a, we count the number of RT villages at different altitudes intervals and display them through a barplot. It can be seen from the figure that with the increase in altitude, the number of RT villages shows a trend from rising to declining. RT villages are mainly located between 1475 m and 3853 m, accounting for more than 89%, and the highest density is found between 2473 m and 3219 m. The peak density of RT villages is not in the lowest altitude areas. One reason for this phenomenon is that the lowest-altitude area of the QTP occupies only small area (each reclassified altitude internal occupies 1%, 2%, 10%, 11%, 15%, 24%, 27%, 10%, respectively). Another reason is that the lowest area of the QTP is in the southern part of Chayu County, south of the Himalayas, which is blocked by the mountains and isolated from the outside world.

Slope has an important restriction on the layout of the village by affecting traffic conditions, landslide hazards, etc. “Slope” tool in ArcGIS is used to extract slope data from DEM and reclassify through natural breaks methods (Figure 6b). By counting the number of RT villages with different slopes, we discovered that 254 RT villages were distributed in the interval with a slope of less than 4°, accounting for approximately 40% of all; 71% of the RT villages were with a slope below 9°. The result shows that the higher the slope, the fewer the number of RT villages, indicating that the distribution of RT villages is negatively correlated with slope.

Aspect data were derived from DEM through “Aspect” tool in ArcGIS. Figure 6c shows the eight categories of aspects: flat, northeast, east, southeast, south, southwest, west, and northwest. We performed an overlay analysis to obtain the number of RT villages on the various aspects (Figure 7c). In general, no obvious clusters were observed in specific aspects. Therefore, RT villages in QTP were evenly distributed in each aspect.

Relief is the difference of elevation between the highest point and the lowest point in a certain area. It is one of the critical indicators of suitability assessment of human settlement as well as resources and environment caring capacity. Referring to the findings of Feng et al. [57], the optimal window size for relief study in QTP is about 1.51 km^2^. We use a rectangular neighborhood of 14 × 14 pixels (about 1.58 km^2^) as the window size and use “Focal Statistics” in ArcGIS to extract the range of elevation in the analysis window. Most RT villages are in moderate and low fluctuation terrain (Figure 6d). Observing the number of RT villages in different relief amplitudes (Figure 7d), 268 RT villages had a relief amplitude between 0 and 200 m, having a proportion of 42%, and the level of 200 m to 442 m accounting for 42%. The number of RT villages with relief amplitude greater than 790 m was only accounting for 1% of the total. Therefore, the layout of RT villages tends to be concentrated in the areas with small or medium relief amplitude, where the settlement conditions, resources, and environment can meet the developmental needs of the RT villages.

#### 3.2.2. Climate

**Figure 8:**
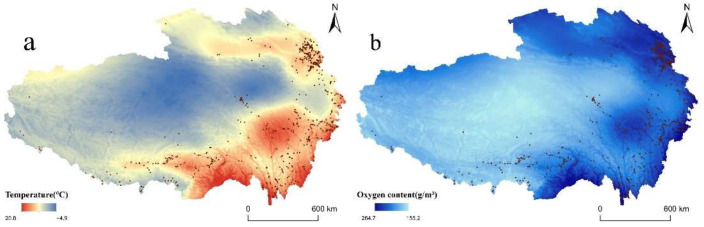
Spatial distribution of RT villages coupled with climate: (**a**) temperature; (**b**) oxygen content.

Low temperature and oxygen shortage are the main climate characteristics of QTP due to its high altitude. Temperature affects the distribution of RT villages by affecting the tourism climate comfort and the villagers’ living environment. As shown in Figure 8a, the average annual temperature in QTP is from −4.9 °C to 20.8 °C. By counting the numbers of RT villages with different temperatures, we can find that between 6 and 8 °C, the number of RT villages is the largest, accounting for 41% of the total. It was followed by 8–10 °C, accounting for 23%. The villages above 10 °C account for 20%, and the villages below 6 °C account for 16%.

Oxygen content in QTP is only 65% of the oxygen content at sea level, which is the main factor restricting human activities. Oxygen content proved to have a strong correlation (R > 0.999) with atmospheric pressure in QTP, so we calculated the oxygen content based on atmospheric pressure data using the formula proposed by Tang [58]. As shown in Figure 8b, average annual oxygen is superimposed with the distribution of RT villages. It can be found that 39% of villages are distributed in an area with an oxygen content of 220–230 g/m^3^. Villages with an oxygen content of 210–220 g/m^3^ and 230–240 g/m^3^ each accounted for 14% of the total. 

#### 3.2.3. Hydrology

**Figure 9:**
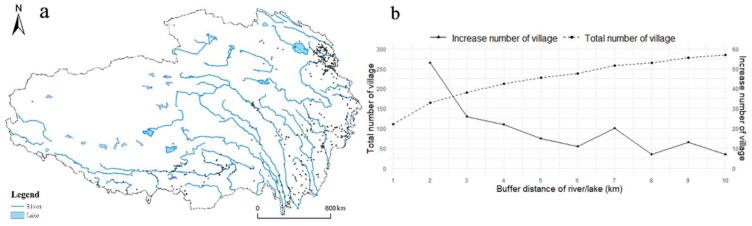
The relationship between river/lake and the RT villages: (**a**) spatial distribution of RT villages coupled with river network; (**b**) the change trend of the number of RT villages with buffer distance of river/lakes.

QTP is the source of the nine largest rivers in Asia, including the Yangtze River, the Yellow River, and the Mekong River, known as Asia’s water tower. The lakes are numerous and widespread, including Qinghai Lake, the largest lake in China. River/Lake is a type of natural landscape resource for tourism and is the primary water source for villagers’ production and life. As shown in Figure 9a, the distribution map of RT villages was superimposed with the river network map in QTP. Further, we use the “Buffer” tool in ArcGIS to count the number of RT villages within different distances from the river/lake. From Figure 9b we can find that the total number of RT villages increases with increased distance from the river/lake. Within the distance of 10 km, the total number of RT villages is 285, accounting for 45%. From the perspective of changing trends, the increase rate of RT villages has slowed down within 2–6 km from the river/lake. Within 6–7 km, there is a relatively high increase rate due to a balance between exposure to risks such as floods and benefits such as access to water. Above 9 km the increase rate of RT villages has dropped again. The distribution characteristics of nearing the water are obvious.

#### 3.2.4. Traffic Condition

Traffic condition is a crucial factor affecting the development of RT through the time and economic cost for tourists and villagers’ ability to obtain resources and connections with the outside world. Roads are the main mode of transport in QTP, we judge the impact of transport accessibility by counting the number of RT villages within different distances of three main road types: highways, national roads, and provincial roads. As shown in Table 2, 94.3% of the RT villages are located within 50 km of roads, and 65% of the RT villages can reach by road within 10 km. From the perspective of road type, provincial road covers most villages within the same distance, which highway covers the least. The spatial distribution of RT villages in QTP depends more on low-grade roads. This is related to the fact that the highway belongs to closed-off management, which controls the entrance and exit, so the influence of the highway will be slightly less than on national and provincial roads. Compared with national roads, provincial roads have longer mileage and broader distribution in QTP, so provincial roads have a more significant impact on the distribution of RT villages.

**Figure 10:**
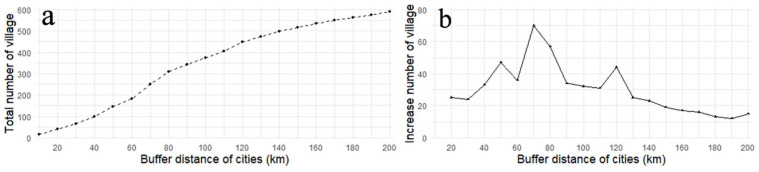
The relationship between the number of RT villages and the bufferdistance of cities: (**a**) total number of RT villages; (**b**) increase number of RT villages.

Cities are the primary source of tourists for RT. We analyze the impact of distance to the source market using the buffer tool to count the number of RT villages within different buffer ranges of prefecture-level administrative center cities in QTP. From Figure 10a, we can find that the total number of RT villages increases with the increase in buffer distance of cities. 58% of total RT villages are within the 100 km buffer zone around the cities, and 92% of the total are within the 200 km buffer zone. From the perspective of the changing trend of RT villages within different buffer distance, we can find that from 0 to 70 km, the increased number of RT villages show a fluctuating upward trend. At 70 km the RT villages formed a dense distribution zone around cities. Above 90 km the increased number of RT villages shows a downward trend.

#### 3.2.5. Socio-Economic 

**Figure 11:**
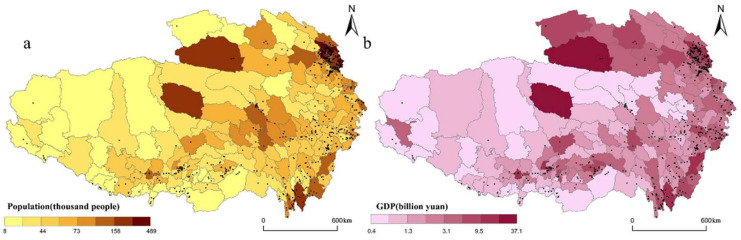
Spatial distribution of RT villages coupled with socio-economic: (**a**) population size; (**b**) the level of economic development.

Based on Figure 11a we find that the RT villages in QTP are mainly concentrated in relative densely populated areas. A Pearson’s correlation analysis of the total population of each county and the number of RT villages in each county found that the coefficient between the total population and the number of RT villages was 0.33, which was significant at a 0.001 level. This shows that the population size and the spatial distribution of RT villages have a medium correlation. The development of RT needs the support of the population.

Gross domestic product (GDP) is an important indicator of economic activity. Figure 11b shows the relationship between the distribution of RT villages and GDP. Further, we use Pearson’s correlation analysis to measure the relationship between GDP and the spatial distribution of RT villages. The Pearson’s correlation coefficient between the GDP of each county and the number of RT villages in each county was 0.14, indicating that the level of economic development and the spatial distribution of RT villages have a small correlation. The higher the level of economic development, the broader the demand for tourism, and the stronger the ability to support the development of RT in the region.

#### 3.2.6. Tourism Resource

**Figure 12:**
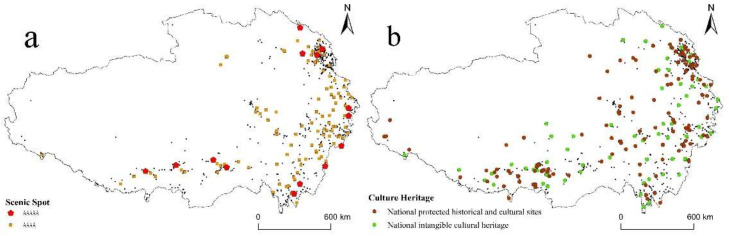
Spatial distribution of RT villages coupled with tourism resource: (**a**) scenic spots; (**b**) culture heritage.

According to the quality rating of the scenic spot system in China, scenic spot was rated from A to 5A for its overall tourism quality. We choose 5A and 4A scenic spots to analyze the impact of scenic spot on the distribution of RT villages. Scenic spots have a radiating and driving effect, which can effectively promote the development of RT in the surrounding villages. As shown in Figure 12a, there is a totally 15 5A and 123 4A scenic spots in QTP. By using buffer analysis, we find that 9 5A scenic spot have more than one RT village within the 10 km buffer range, and 74 4A scenic spots have more than one RT village within the same buffer range. RT villages tend to be located in areas with rich scenic spots.

QTP is with multi-ethnic, multi-religious, multicultural diversity, and rich cultural heritage. We use National protected historical and cultural sites, and National intangible cultural heritage of China to analyze the relationship between culture heritage and RT villages. According to Figure 12b, there are 166 National protected historical and cultural sites, and 264 National intangible cultural heritage in QTP. Using Pearson’s correlation analysis, the result shows that the coefficient between the number of total cultural heritage in each county and the number of RT villages in each county is 0.22, which was significant at a 0.01 level. Therefore, there is a low degree positive correlation between cultural heritage and the distribution of RT villages.

### 3.3. Driving Mechanism

We explore the explanatory factors from a spatial perspective and the potential interactive impacts of these factors. We use the factor detection module and the interaction detection module in Geodetector to analyze the driving factors of the spatial distribution of RT villages. According to Equation (6), we set the kernel density value of RT villages as dependent variable Y, and twelve evaluation indexes of six influencing factors shown in Table 3 as independent variable X. First, the study area was spaced at 5 km intervals, and 125,175 sampling points were generated to sample kernel density value and twelve continuous type independent variables. Then equivalence breakpoint method, natural break method, quantile method, geometric break method, and standard deviation method were used as statistical stratification methods with intervals of 3–6. Finally, the scheme with the enormous Q-statistic was selected as the stratification and interval parameters for twelve evaluation indexes [59]. 

Table 3 shows the factor detection result of 12 indexes. The results contain two values, Q-stastic represents the explanatory power of the index and the *p*-value represents the significance of this index. Since the *p*-value for all 12 indexes are 0.000, all indexes are significant. From the perspective of the explanatory power of indexes, population size (0.333) and scenic spot (0.332) have the highest explanatory power, implying that the distribution of RT villages is closely related to the distribution of population and scenic spots. People are the mainstay of tourism activities, and areas with a large population size have a wider tourist source market and greater potential for tourism development. Scenic spots are the core of tourism activities, villages near scenic spots can benefit from the scenic spot and are more likely to meet the threshold for RT development. Oxygen content (0.242), Major cities (0.237), Culture heritage (0.237), and Temperature (0.224) have the same level of high explanatory power, which meant that these four indexes have a noticeable impact on the distribution of RT villages. GDP (0.176) and Altitude (0.131) also influence the spatial distribution. Main roads (0.092), River/lake (0.059), Slope (0.054), Relief (0.042) have minimal explanatory power on spatial distribution of RT villages. In terms of influence factors, the rank of Q-statistics is: Tourism resource (0.569) > Socio-economic (0.509) > Climate (0.466) > Traffic condition (0.329) > Topographic (0.227) > Hydrology (0.059). Therefore, tourism resource and socio-economic factors are the prominent driving forces of the distribution of RT villages. Climate, traffic, and topographic conditions also have a certain impact on RT villages to different extents.

**Figure 13:**
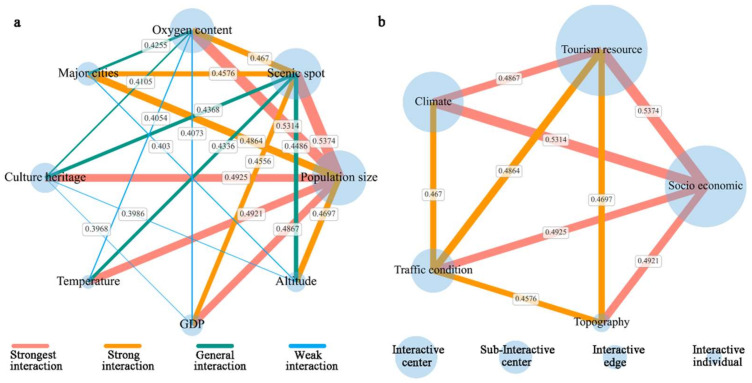
Diagram of interactions network: (**a**) evaluation indexes; (**b**) influencing factors.

In order to further explore the interaction mechanism of factors on the spatial distribution of RT villages, we conduct the interaction detection of evaluation indexes. The result generates 66 interaction combinations of evaluation indexes and their interaction Q-statistic. We find that the interaction Q-statistic for all combinations is greater than the Q-statistic for individual indexes. Fifteen of the interaction combinations are nonlinear enhancements, in which the Q-statistic for interaction is greater than the sum of the two indexes; the remaining 51 combinations are bi-enhancement, in which the Q-statistic for interaction is greater than the maximum of two indexes. It denoted that each combination of indexes has bilinear or non-linear enhancement and mutually reinforcing effects on forming RT villages. Then we filtered twenty combinations with the maximum Q-statistic values from the 66 interaction combinations. These twenty combinations play a vital role in the spatial distribution of RT villages. As shown in Figure 13, we mapped the evaluation indexes as nodes and the interactions between indexes as edges. The width of the edge is determined by the Q statistic for the interaction between indexes, and the size of the node is measured by the cumulative value of the Q statistics for the interactions between the node and other nodes. From the perspective of the interaction of evaluation indexes, the strongest explanatory power was found between the interaction of population size and scenic spot (0.537), which is significantly higher than driven by a single index, and the influence power of interaction between population size and other indexes are also more significant. This suggests that population is the core of RT development in QTP. Scenic spot as a key index also has strong interaction with other indexes (>0.4), this reflects the fact that scenic spot greatly enhances the overall impact of the combination with the other indexes. In terms of influencing factors, the spatial distribution of RT villages results from multifactor interactions. Socio economic and tourism resource are dominant factors in this complex mechanism network, climate is the key factor, traffic condition is the guarantee factor, and topography is the foundation factor.

## 4. Discussion

### 4.1. Comparison of the Results of Related Studies

Regarding the spatial distribution character, the distribution of RT villages on the QTP is similar to the distribution of RT villages in other regions of China, showing an aggregated distribution character. Moreover, the distribution is generally characterized as dense in the southeast and sparse in the northwest, taking the Hu line as the boundary (Qilian-Jilong Line is part of Hu line). Simultaneously, the nearest neighbor index of the QTP (0.39) is lower than those of China as a whole (0.52) and Shanxi (0.9), Liaoning (0.82), Jiangsu (0.54), and Hubei (0.62) provinces, reflecting that the spatial distribution of RT villages on the QTP is more concentrated than in other parts of China. We compared the distribution of RT villages on the QTP under different influencing factors with the distribution of RT villages nationwide. In terms of topographical factors, the altitude of the RT villages on the QTP (2470–3220 m) is significantly higher than the altitude of the RT villages nationwide (<500 m), and the slope on the QTP (<9°) is slightly higher than nationwide (<6.7°). In terms of hydrological factors, the number of RT villages decreased with the increase in the distance from the river and lake both in the QTP and nationwide. In terms of traffic condition, the proportion of RT villages on the QTP is greater than the proportion of RT villages nationwide within the same distance from roads. However, the proportion of RT villages on the QTP is smaller than the proportion of RT villages nationwide within the same distance from cities. Regarding tourism resource, the impact of scenic spots on the RT villages nationwide is close to that of the QTP. 

### 4.2. How to Develop RT Villages in QTP

Based on a comprehensive analysis of the spatial distribution characteristics, influencing factors, and driving mechanism of RT villages, we propose the following scientific suggestions for the sustainable development of RT in QTP, helping to achieve the goal of green development and making QTP a world tourism destination.

(1) Improving regional RT village structures and promoting coordinated regional development. The current spatial distribution of RT villages which are spatially concentrated and highly centralized represents the choice of the tourism market. However, this will hinder the balanced and sustainable development of the RT industry in the long term. Some areas will be overdeveloped and lead to relatively fierce competition in the later stages, and some areas will result in the decay of existing RT resources through disuse. To avoid or reduce this phenomenon, the government should play a greater role to ensure more balanced and coordinated development. Huangshui Valley as the most densely distributed area of RT villages, should unleash the agglomeration effect and spillover effect, and focus on the improvement of tourism quality and guiding the market to reduce unnecessary repetitive construction. YNL River region as a relatively un-dense distribution area should scale up financial support to exploit more tourism resources and increase policy support for inscribing on various RT villages lists.
(2) Strengthen ecological and environmental protection. The special geological, topographic, and weather conditions on the QTP make its ecosystem fragile and highly vulnerable to environmental changes [60]. Moreover, the ecological environment of QTP is important to the sustainable development of East Asia and the whole world. So, it is important to protect the ecological environment of the QTP. Tourism will affect heavily the environment of QTP if not properly managed. Along the needs for transport, food and accommodation will exert enormous stress on the ecology of QTP. Therefore, it should coordinate the relationship between RT development and environmental protection, take the natural ecological environment of the village as an important attraction for RT development, and create a green and livable RT destination. To achieve this, first, the size of RT villages and the number of tourists should depend on the tourism environment carrying capacity of the village. Second, any tourism-related infrastructure in the village must be environmentally sustainable. Finally, governments need to increase efforts to raise environmental protection awareness of local villagers and tourists.
(3) Supporting the integration of culture and tourism. The sense of the holy land, the feeling of purity, and the mysterious atmosphere of religion distinguish QTP rural areas from the cultural atmosphere in other regions. The cultural resources of the QTP include ethnic cultural resources, mainly Tibetan, Han, Qiang, Hui, and other ethnicities, and religious cultural resources, mainly Tibetan Buddhism, Islam and Taoism, and folk culture resources, mainly costumes, catering, etiquette, and festivals, digging into the cultural connotation and preserving, inheriting, and promoting the culture of QTP. First, adhering to the principle of combining conservation and development of culture. The cultural differences between tourists and villagers will cause a huge impact on the villagers. It is important to assess scientifically, protect primarily, and develop appropriately, so that the rich and colorful cultural resources of the QTP can be developed sustainably. Second, highlighting the different regional characteristics and features of the culture, showing the diversity of QTP cultural resources. Generally, QTP can be divided into frontal Tibet, interior Tibet, An Tibet, and Kang Tibet. The culture differs significantly from region to region. The development of cultural resources in each region should complement and be independent of each other. Last, encouraging community involvement. Villagers are both creators and owners of culture. The participation of the local community is not only conducive to increasing the income, but also to maintaining the original local character.
(4) Taking the active steps to respond to climate change. QTP shows a large-scale warming trend of 0.3 °C per decade since the mid-1950s, which is significantly three times faster than the global warming rate. Climate change has caused glacier retreat, snow cover change, and permafrost degradation of QTP [61], and may have implications for wetland-based tourism, ski-based tourism, glacier-based tourism, plant-based tourism, and wildlife-based tourism of QTP [62]. Climate change is a real threat to many rural regions which affects the RT offerings. As Arunima Karali suggested, climate change should incorporate in the context of sustainable RT because dealing with climate change issues has been a prerequisite in the sustainable tourism research. Therefore, we should consider RT strategy and management to try to mitigate climate change challenges and support rural community resilience. First, the government should establish a climate monitoring and early warning platform, (i) providing severe weather forecasting; (ii) providing tourism climate comfort relate information; (iii) providing phenology relate data, guiding flower-viewing rural tourism and picking rural tourism. Second, the government should conduct awareness and education to improve the perception of climate change risks of all stakeholders. Encouraging visitors to adopt “climate-friendly” consumption habits, such as consuming local food instead of foreign food, and reducing their carbon footprint. Providing publicity on scientific knowledge related to coping with extreme weather to local villagers, to enhance awareness of disaster resilience and response.

### 4.3. Limitations of the Study

Despite the analysis of spatial characteristics, influencing factors, and driving mechanisms of RT villages on QTP has contributed to the sustainable development of RT in the area, some limitations should be addressed in the future. (1) Due to the limitation of data, we could not obtain detailed information of 640 RT villages. So, this paper analyzes RT villages as point elements, which fails to classify and assign weights of RT villages based on their market size, tourism product, and profitability. In future research, it will be necessary to explore in depth the spatial distribution of different types of RT villages. (2) The influence factors focus mainly on the macroscale including physical and socio-economic factors. However, it lacks the microelements, such as village economic conditions, agricultural production, villagers’ tourism perception, and villagers’ tourism participation willingness. These factors would be worthy of further research. (3) This article focused on the study of RT villages from the spatial perspective and lacks research from the temporal perspective. In future, sequential time-series data can be supplemented to analyze the spatio-temporal evolution of the RT villages and to make judgements about future trends.

## 5. Conclusions

Analyzing the spatial distribution characteristics, influencing factors and driving mechanism of RT villages have important theoretical and practical implications for the development and growth of RT in QTP. The main conclusions of the study are as follows:

1. The overall spatial distribution of RT villages in the QTP is dense in the southeast and sparse in the northwest, taking Qilian-Jilong Line as the boundary. The nearest neighbor index is 0.39, indicating that RT villages show aggregated distribution character. The uneven spatial distribution of RT villages is significant at the inter-county level, in which the geographic concentration index is 13.3%, and the Lorentz curve presents a typical concave form. Regarding spatial pattern, RT villages have formed four relatively dense distribution areas, namely the Huangshui Valley, West Sichuan Mountain, Northwest Yunnan and YNL River region. RT villages in QTP have a positive spatial autocorrelation (Moran’s I = 0.268), and 59.1% of the area belongs to cold spots and 2.13% of the area belongs to hot spots based on the cluster of correlation.
2. The analysis of topographic factors revealed that medium altitude, low slope, and moderate fluctuation had become the main areas for the distribution of RT villages. Regarding climate factors, 84% of RT villages have an annual temperature greater than 6 °C, and 58% of the RT villages have an oxygen content greater than 220 g/m^3^. In terms of hydrology, the spatial distribution of RT villages shows hydrophilic characteristics, 45% of RT villages located within 10 km from rivers or lakes, and the number of RT villages decreasing with the increased distance from the rivers or lakes. From the perspective of traffic condition, 65% of the RT villages are accessible by road within 10 km, with the most accessible by provincial road and the least accessible by highway. Moreover, 58% of RT villages are located within 100 km of central cities. Concerning the distribution of socio-economic factor, the population size has a medium correlation with the RT villages (r = 0.33), and the level of economic has a small correlation with the RT villages (r = 0.14). As for tourism resource, the spatial distribution of RT villages is positively related to the layout of scenic spot (r = 0.31) and culture heritage (r = 0.22).
3. The spatial distribution of RT villages is the result of multifactor interactions. From the single driving factor, the factor with the strongest explanatory power is population size (0.333) > scenic spot (0.332) > oxygen content (0.242) > major cities (0.237) > culture heritage (0.237). In terms of the composite factor interaction results, the explanatory power of the interaction is better than the single factor, the interaction network formed by tourism resource and socio economic strongly determines the spatial distribution pattern of the RT villages.

## Figures and Tables

**Table 1:** Influencing factors of spatial distribution of the RT villages.

Influence Factor	Evaluation Index	Geographical Proxy Variables
Topographic	Altitude	Altitude
	Slope	Slope
	Aspect	Aspect
	Relief	Relief
Climate	Temperature	Average annual temperature
	Oxygen content	Average annual oxygen content
Hydrology	River/Lake	Distance from river/lake
Traffic Condition	Transport access	Distance from main roads
	Distance to source market	Distance from central cities
Socio-economic	Population size	Total population
	Level of economic development	Gross regional product
Tourism resource	Scenic spot	Distance to high level scenic spots
	Culture heritage	Distance to culture heritage

**Table 2:** The relationship between the number of RT villages and buffer range of different types of roads.

	10 km	20 km	30 km	40 km	50 km
Highway	130	214	255	291	315
National road	169	224	273	306	342
Provincial road	262	352	443	489	532
All road	416	500	563	587	604

**Table 3:** Geographical detection results of factors influencing the spatial distribution of RT villages.

	Altitude	Slope	Relief	Temperature	Oxygen Content	River/Lake
Q-stastic	0.131	0.054	0.042	0.224	0.242	0.059
*p*-value	0.000	0.000	0.000	0.000	0.000	0.000
	**Main Road**	**Major Cities**	**Population**	**GDP**	**Scenic Spot**	**Culture Heritage**
Q-stastic	0.092	0.237	0.333	0.176	0.332	0.237
*p*-value	0.000	0.000	0.000	0.000	0.000	0.000

## Data Availability

Not applicable.
